# The impact of practical experience on theoretical knowledge at different cognitive levels

**DOI:** 10.4102/jsava.v91i0.2042

**Published:** 2020-07-29

**Authors:** Rhoda Leask, Tanita Cronje, Dietmar E. Holm, Linda van Ryneveld

**Affiliations:** 1 Department of Production Animal Studies, Faculty of Veterinary Science, University of Pretoria, Pretoria, South Africa; 2 Department of Statistics, Faculty of Natural and Agricultural Sciences, University of Pretoria, Pretoria, South Africa; 3 Department of Comprehensive Online Education Services, Faculty of Education, University of Pretoria, Pretoria, South Africa

**Keywords:** education, small ruminants, veterinary graduates, curriculum design, practical experience, theoretical education, assessment

## Abstract

Although theoretical training of veterinary students is uncomplicated even for larger groups, practical training remains a challenge. Much has been said about the value of practical training in curriculum design. Yet, the impact of practical training on theoretical knowledge needs further research. A cohort of 89 students with very limited clinical practical experience completed an assessment at the end of their theoretical training in small ruminants. The scores obtained by the students were compared with those obtained by a group of 35 veterinarians who volunteered to participate in the study. In addition to comparing the scores between students and practitioners, the cognitive level of each of the questions was considered. Overall, veterinarians achieved higher test scores than did the students. The veterinarians outperformed the students in all cognitive levels except for ‘applying’ type questions where there was no difference. Different levels of experience, namely young veterinarians (*n* = 11), established veterinarians (*n* = 13) and veterinarians approaching retirement (*n* = 11), were evaluated against the revised Bloom’s cognitive levels. When modelling congress attendance frequency, years’ experience, proportion of time spent with ruminants and revised Bloom’s levels, congress attendance was not a significant variable, and thus, only the other three variables remained. This investigation found that practical experience has a positive effect on theoretical knowledge. The type of practical experience and where such practical experience is included in a curriculum need further research. Working for a number of years in a specific discipline will provide the best support for theoretical knowledge.

## Introduction

An increase in the use of technology has prompted an increase in undergraduate veterinary student numbers. At the University of Pretoria’s Faculty of Veterinary Science, intake figures remained the same from 1976 until 2001 when there was an increase of 33%, followed by further increases of 13%, 11% and 27% in 2006, 2011 and 2014, respectively. Collins and Taylor (2002) and Allworth (2014) believed that undergraduate training would continue to focus on generalised knowledge. As a result, there would be an increase in demand on postgraduate training to develop practical skills, particularly in ruminants. This in turn has resulted in the development of postgraduate training centres such as the recent development of the European College of Small Ruminant Health Management (Bath et al. 2006; Fthenakis 2008). Katajavouri, Lindblom-Ylänne and Hirvonan (2006) stated that today’s experts have to be able to refresh their expertise on a continual basis. This is already a requirement in the veterinary profession to maintain registration and is referred to as continuing professional development (CPD). This needs to be done to apply the knowledge acquired into practical work and to be updated with current knowledge and practice, and it is particularly important when considering the technological advances in today’s society.

Formal theoretical learning is essential for expert knowledge (Katajavouri et al. 2006). Although Katajavouri et al. (2006) believed that informal practical knowledge (or skills development) is learnt in the workplace, others see the importance of integrating it into the curriculum as part of the final year programme (Kiggundu & Nayimuli 2009; Irons, Holm & Annandale 2017; Walley & Albadri 2015). Irons et al. (2017) emphasise that this is important in producing a Day One Competent veterinarian, and all the competencies expected in the curriculum are included as they are considered Day One Competencies thereby allowing a new graduate to practise effectively on the first day. There are, however, those who doubt the value of practical experience (Hodson 1990; Osbourne 1993; Woolnough & Allsop 1985), and it is true that the type of practical experience, and not just any practical experience for the sake of inclusion in a curriculum, is important (Pienaar 2014). An effective way of integrating practical experience as part of an undergraduate degree is workplace-integrated learning (WIL). Pienaar (2014) also referred to as real-world learning as discussed by Wrenn and Wrenn (2009). It is important to allow students to do the practical work involved in WIL with as little interference by the professional as possible to gain the maximum benefit of practical training (Wrenn & Wrenn 2009). However, this is often difficult in the medical fields where lives as well as the professional’s practice reputation are at stake.

Eraut (1994) stated that theoretical knowledge alone could not prepare students for the challenges faced in working life. Although Benner, Tanner and Chesla (1995) suggested that practical and theoretical knowledge support the application and use of one another, Millar (2004) mentioned that practical work includes interpretation of data and that learning involved in practical activity mostly occurs through discussing observations and measurements and interpreting them.

Katajavouri et al. (2006) further stated that metacognitive skills are acquired through practical experience and are important for lifelong learning. Lifelong learning is an integral part of the veterinary profession. It has been confirmed that practical knowledge is contextual, and it is important for students to understand the link between theory and practice to apply theoretical knowledge in the workplace (Katajavouri et al. 2006).

Walley and Albadri (2015) reported that in a survey, final year dental students in the United Kingdom with more practical experience felt more capable of discussing inhalation sedation with patients and parents and were more satisfied with the quality of teaching. Increased student satisfaction with training was interpreted by Walley and Albadri (2015) as owing to more practical training being included in the curriculum. Students in the pre-clinical years at the Faculty of Veterinary Science of the University of Pretoria experience limited and basic practical training. The majority of the practical training occurs in the final year and a half of the current curriculum (Irons et al. 2017). Kiggundu and Nayimuli (2009) found that student teachers viewed teaching practice as an important component of their training. They reported that it assisted in contextualising the theoretical knowledge. However, the study on the dentistry students was perception-based. There are a number of ways that questionnaires may be compiled (Bailey 1978; Berdie 1973; Berdie, Anderson & Niebuhr 1986; Montgomery & Crittenden 1977; Sheatsley 1983) and interpreted (Allen & Seaman 2007; Clason & Dormody 1994). The order of the questions is important (McClendon & O’Brien 1988), and such questionnaires can include open-ended questions (Geer 1988; Perkin 1995) and Likert scales (Gliem & Gliem 2003; Likert 1932). Although questionnaires (when set out correctly) and their use may provide valuable information, quantitative data on a subject can provide alternative perspectives. Thus, the authors hypothesised that it would be relevant to test the impact of practical training in a quantitative way as performed in this study.

The study by Katajavouri et al. (2006) confirmed that practical knowledge is contextual. It is therefore important to understand the link between theory and practice to apply theoretical knowledge in the workplace. Their study concluded that it is important to include practical training at an undergraduate level to ensure that students can recognise the need for certain theoretical components of a degree and demonstrate how these theoretical components can be applied practically.

If practitioners perform better at an assessment based on theoretical knowledge taught prior to the clinical year, than the students (who have recently completed the formal theoretical training with the knowledge fresh in their minds), then there is a strong motivation to incorporate more practical training into earlier years of the curriculum. In so doing, a more capable Day One Competent (Irons et al. 2017) veterinarian can be produced.

This study aims to determine what effect practical training has on theoretical knowledge and the ability of students or veterinarians to apply such practical training to answer theoretical questions in a computer-based assessment as currently used to assess pre-clinical (fifth year) veterinary students. In other words, to determine whether the converse of what all these studies have concluded can be applied, by proving that practical experience can reinforce theoretical knowledge and allow it to be better applied.

## Materials and methods

### Material studied

An assessment consisting of 90 questions totalling 101 marks was compiled as a standard computer-based assessment used for formative assessment in the pre-clinical (fifth) year of the Bachelor of Veterinary Science degree at the University of Pretoria. Quality control by the education innovation and academic staff was implemented to categorise the questions according to Bloom’s Taxonomy (Bloom et al. 1956) as revised by Anderson and Krathwohl (2001) with the following outcome: Remember (*n* = 18), Understand (*n* = 22), Apply (*n* = 23), Analyse (*n* = 22) and Evaluate (*n* = 5). The data were classified as ordinal data as described by Allen and Seaman (2007). The allocation of the Bloom’s categories was thus 80% in the higher-order thinking range and 20% consisting of lower-order thinking questions. Nine of the 90 questions (10%) were multiple response questions and the rest were multiple choice questions. Two academic staff members then reviewed the article and memorandum for correctness. The questions were also categorised into topics to determine whether students had experience with a specific topic. Topics included biosecurity, economics, ectoparasites, internal parasites, lameness, management, nutrition, pathology, the perinatal period, reproduction, respiratory conditions, selection and culling, skin conditions, sudden deaths, vaccines and zoonoses.

Questionnaires were set for the participants (both veterinarians and students) to determine their level of practical experience. Studies by Berdie (1973), Montgomery and Crittenden (1977), Bailey (1978), Sheatsley (1983) and Berdie et al. (1986) were used to draw up the questionnaire according to evidence-based methods. Open-ended questions were also included as suggested by Geer (1988) and Perkin (1995), and the order of questions was considered in accordance with suggestions by McClendon and O’Brien (1988). Most questions were classified as ordinal data, interval data and ratio data (Allen & Seaman 2007) with some open-ended questions that were coded and analysed separately.

### Participants

Those who were willing to participate (students and veterinarians) in the study signed a letter of informed consent. This letter informed them of their rights during the study and of the expected outcomes of the study. The researchers worked with a convenience sample of willing participants. For future studies, it would be recommended that power analysis be performed before the study commences. However, a power analysis was performed on the assessment that compared the scores between the veterinarians and the students, using G*Power 3.1.9.2, at an alpha level of 5%, and a large effect size. The power analysis showed that the sample sizes of both groups were large enough to ensure a power of above 90%.

#### Veterinarians

Rural practitioners and faculty members were approached. Some were approached personally in addition to a call for participation on a local information platform ‘ruralvet’ (ruralvet@yahoo.com). Some practitioners passed the assessment to colleagues as well.

Thirty-five of the 42 respondents provided useable data. Those excluded had incomplete responses as entire sections were omitted from the survey and the assessment. However, of the 35, some had failed to answer one or two questions that could be dealt with as missing data that will not affect the outcome of calculations as described by Dohoo, Martin and Stryhn (2003), where data are excluded from the calculations. Respondents included private veterinarians, state veterinarians and practising veterinarians in the ruminant health industry.

#### Students

The student cohort that were registered for a small ruminant pre-clinical (fifth year) module described by Pettey (2014) were included in the study. There were 163 students registered for the module, and 89 of the 116 students who agreed to participate provided useable data, as they attended the necessary classes and completed the assessment without too many missing values.

### Procedure

The Research and Animal Ethics Committee of the University of Pretoria approved the study (V018-17). To reduce resistance to participation by busy rural practitioners, the assessment was sent to the veterinarians with a ‘self-imposed’ time limit of 2 hours to complete the assessment and questionnaire. There was no time restriction, as it is understood that practising veterinarians may not have two consecutive hours to set aside for completing the task and some would have to leave the assessment mid-way to attend to cases before returning to complete the task. The students were required to sit the assessment as per the University of Pretoria rules and regulations. Students were given 2 h to complete the assessment, and the order of the questions for the students was randomised. Having completed the assessment, students were given additional time to complete the questionnaire. The veterinarians were asked not to spend too much time looking up answers, but to base most of their answers mainly on what knowledge they had acquired through their studies and experience. However, because it was difficult to monitor whether they spent much time referencing answers, the assessment was regarded as ‘open resource’, thus allowing for both the students and the veterinarians to look up answers on the internet, textbooks or class notes.

The veterinarians received the questions in the same order during the assessment as it was emailed as a document, where they could indicate their selection. The students completed the assessment in the Faculty’s computer laboratory and hence received the questions in a randomised order as is commonly done at the Faculty to prevent group polarisation where one person may influence the answers of those around him or her as found by Myers and Lamm (1976).

The following information was used from the questionnaires to determine the amount of practical experience:

- Number of years’ experience (veterinarians)
- Time spent with ruminants (veterinarians)
- Congress attendance frequency (veterinarians)
- Practical experience with any of the topics (students)

Number of years’ experience was classified as interval data, although time spent with ruminants was classified as ratio data (Allen & Seaman 2007). Congress attendance frequency and practical experience for the students were classified as ordinal data. The congress attendance categories for the veterinarians were assigned as follows: missing data (0), less than once every 4 years (1), once every 4 years (2), once every 3 years (3), once every 2 years (4) and once a year (5). The student practical experience was assigned according to the number of topics that the students had experienced in a practical way – mostly through observation of cases. Only eight students had this type of experience, mostly through observation. This was not considered to be of clinical experience. It was further investigated whether any practical experience obtained in earlier years of the degree would have had an effect on marks obtained for the current assessment. To do this, student marks obtained for the small ruminant section of a second year module, which included practical handling of sheep and goats and farm procedures, were compared to the marks obtained for the current assessment. There was no significant correlation in the marks (*p* = 0.85) using Fisher’s Z transformation (Fisher 1915), and therefore, all students were allocated 0 years’ clinical experience.

It was then determined whether the cognitive level of questions had an effect on students’ and veterinarians’ test scores. Finally, to determine whether there was a difference according to the number of years’ experience, the veterinarians were then categorised into three groups. They were categorised as follows: qualified veterinarians with less than 6 years’ experience (young veterinarians, *n* = 11), veterinarians with 6–31 years’ experience (established veterinarians, *n* = 13) and veterinarians with more than 31 years’ experience (approaching retirement veterinarians, *n* = 11).

### Data analyses

The Shapiro–Wilk’s test is one of the most popular tests for normality assumption diagnostics and was used to evaluate if the data within the groups being compared were normally distributed or not. It was found that only half of the groups’ data was normally distributed. Because the normality assumption of the parametric tests was not met, the non-parametric alternative test, the Mann–Whitney U test, was used to compare the test scores between the veterinarians and students (hence also reporting the median and interquartile range [IQR]), for each of the cognitive levels (Rani Das & Rahmatullah Imon 2016; Shapiro & Wilk 1965). The Spearman’s rank correlation was used (which measures the monotonic association between variables) because one of the variables was ordinal in nature rather than the normally distributed continuous data. When comparing correlations and determining whether they differ significantly, the Fisher’s Z transformation was used (Fisher 1915).

A linear regression model was used to investigate the effect that congress attendance frequency, the number of years’ experience, time spent with sheep and goats and the revised Bloom’s level had on the veterinarians’ test scores. As the students did not contribute to all the variables in the model, their data were excluded from the model, and only the veterinarians’ data were used.

### Ethical consideration

Ethical approval was obtained from the Research and Animal Ethics Committee of the University of Pretoria (V018-17).

## Results

Overall, the students achieved lower scores than did the veterinarians (median, IQR: 51.5, 47.5–51.5 vs. 62.4, 55.5–62.4, *p* < 0.01). This was the case for all cognitive levels, except for applying.

When comparing each subgroup of veterinarians with the student scores, young veterinarians’ scores did not differ from students’ scores for higher cognitive level questions, whereas this was not the case for more experienced veterinarians (Table 1).

**TABLE 1:** Comparison of students’ scores to three categories of experienced veterinarians’ scores for the different revised Bloom’s levels.

Cognitive level	Students (*n* = 89)		Young vets (*n* = 11)			Established vets (*n* = 13)			Approaching retirement vets (*n* = 11)		
	Median	IQR	Median	IQR	*p*	Median	IQR	*p*	Median	IQR	*p*
Remembering	62.5	54.2; 70.8	76.9	57.4; 84.3	0.04*	71.3	58.3; 80.6	0.08	82.4	79.6; 88.0	< 0.01*
Understanding	50.0	45.5; 59.1	61.4	52.3; 68.2	0.01*	65.9	56.8; 73.8	< 0.01*	65.9	61.4; 75.0	< 0.01*
Applying	56.5	47.8; 65.2	54.4	43.5; 63.0	0.55	47.8	41.3; 63.0	0.21	63.0	54.4; 73.9	0.09
Analysing	47.8	39.1; 56.5	59.1	47.7; 63.6	0.07	52.3	45.5; 63.6	0.20	70.5	59.1; 73.8	< 0.01*
Evaluating	20.0	20.0; 40.0	40.0	0; 60.0	0.46	40.0	40.0; 60.0	< 0.01*	60.0	40.0; 80.0	< 0.01*

* , Significant *p*-value.

**FIGURE 1:**
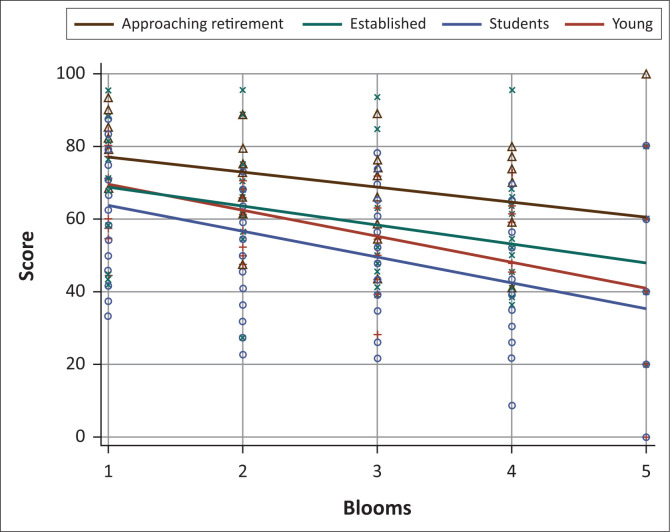
Assessment scores obtained for students, young veterinarians, established veterinarians and veterinarians approaching retirement according to cognitive level.

The correlations between cognitive level of questions and score achieved were −0.55, −0.44, 0.44 and −0.37 for students, young veterinarians, established veterinarians and veterinarians approaching retirement, respectively (*p* < 0.01) (Figure 1). These four correlations were not significantly different from each other.

Nine of the 17 topics resulted in the students achieving significantly lower scores than the veterinarians, the exceptions being internal parasites, mastitis, the perinatal period, respiratory conditions, selection and culling, skin conditions and zoonoses, where there was no difference and vaccines where the students significantly outperformed the veterinarians (Table 2). The results were categorised into topics to determine whether practical experience in a topic assisted students.

**TABLE 2:** Results comparing veterinarians’ to students’ scores within topics.

Topic	Number of questions	Students (*n* = 89)		All veterinarians (*n* = 35)	
		Median	IQR	Median	IQR
Biosecurity	5	60.0†	40.0; 60.0	80.0††	60.0; 100.0
Economics	5	20.0†	20.0; 40.0	40.00††	20.0; 60.0
Ectoparasite	2	75.0†	50.0; 75.0	75.00††	50.0; 75.0
Internal parasites	9	66.7†	55.6; 77.8	66.7†	55.6; 77.8
Lameness	5	40.0†	20.0; 60.0	40.0††	40.0; 60.0
Management	8	33.3†	33.3; 44.4	66.7††	44.4; 66.7
Mastitis	2	50.0†	50.0; 100.0	100.0†	50.0; 100.0
Nutrition	16	47.1†	41.2; 58.8	64.7††	58.8; 76.5
Pathology	7	57.2†	42.9; 71.4	71.4††	57.1; 85.7
Perinatal period	3	50.0†	25.0; 75.0	50.0†	25.0; 75.0
Reproduction	4	50.0†	25.0; 50.0	75.0††	50.0; 75.0
Respiratory conditions	1	100.0†	0.0; 100.0	100.0†	100.0; 100.0
Selection and culling	2	50.0†	50.0; 100.0	50.0†	50.0; 100.0
Skin conditions	4	50.0†	50.0; 75.0	50.0 †	25.0; 75.0
Sudden death	9	66.7†	55.6; 77.8	77.8††	66.7; 88.9
Vaccines	7	71.4†	57.1; 85.7	57.1††	28.6; 71.4
Zoonoses	1	66.7†	66.7; 66.7	66.7†	66.7; 66.7

†, †† , Medians with different superscripts in rows differ significantly (*p* < 0.05).

When modelling the congress attendance frequency, the number of years’ experience, proportion of time spent with ruminants and revised Bloom’s levels, congress attendance was not a significant variable and thus only the three remained (Table 3). The *R*-squared value for the model is 0.3398.

**TABLE 3:** Linear regression of variables against the veterinarians’ scores.

Variable	Coefficient (*β*)	*p*
Intercept	66.61	< 0.01
Number of years’ experience	0.25	< 0.01
Proportion of time spent with sheep and goats	0.20	< 0.01
Revised Bloom’s level	−5.24	< 0.01

## Discussion

Overall, the veterinarians outperformed the students in the assessment. This can be attributed to the fact that qualified veterinarians have clinical practical experience, as opposed to students who were considered to have very limited clinical practical experience. When comparing scores according to the revised Bloom’s levels the veterinarians’ scores were higher than the students’ scores for all the revised Bloom’s levels, all being significantly higher except for the applying level. However, after the veterinarians were categorised into the three groups (young veterinarians, established veterinarians and those approaching retirement), it can be seen in Table 1 and Figure 1 that the cognitive level (the revised Bloom’s level) had a definite effect on all groups, with students and young veterinarians being the most affected groups.

The young veterinarians outperformed the students in remembering and understanding (lower cognitive level), but not at the higher cognitive levels. The established veterinarians outperformed the students in the understanding and evaluating levels (Table 1). This indicates that more experienced veterinarians were better able to apply a higher cognitive level of thinking when answering those types of questions. They could not necessarily remember theoretical knowledge better, but they showed a better understanding of the theoretical knowledge. Those veterinarians approaching retirement were able to outperform the students at all levels, except for applying theoretical knowledge, and their scores ultimately influenced the scores when all veterinarians were combined. It is clear that veterinarians with more clinical practical experience are able to operate at a higher cognitive level than the students with no clinical practical experience (Table 1). It can therefore be concluded that it would be beneficial to include as much practical experience as possible in an undergraduate curriculum and careful decisions must be made in the curriculum design process on the type of practical experience and where to include it in the degree (Posner & Strike 1976; Toohey 1999).

It is important to note that as the assessment was set as a simulated examination paper, not all topics received equal attention, and thus, the number of questions per topic was not the same (Table 2). For the topic analysis, respiratory conditions and zoonoses only had one question each (these questions could not be categorised into another topic and were therefore analysed in their respective topics) as seen in Table 2. Hence, the outcome for these topics was either correct (all veterinarians got the respiratory question correct) or incorrect (some students answered incorrectly; thus, the IQR was 0% – 100%). The students and veterinarians performed the same for the zoonoses, internal parasites, mastitis, perinatal conditions, and selection and culling. There was no difference for skin conditions; however, the range was larger for the veterinarians (IQR 25–75) than the students (IQR 50–75) (Table 2). With these topics, an explanation for the veterinarians not outperforming the students can be that they do not encounter these cases on a regular basis as part of their practical experience. This could be clarified by a follow-up questionnaire for further research. One veterinarian commented that some topics were covered more thoroughly than others were during lectures, and this could also explain the discrepancy.

From the model in Table 3, it can be seen that the number of practical years of experience and time spent with sheep and goats had the largest effect on the veterinarians’ scores. For each year of more experience, the scores increased by 0.25%. For every percentage increase in the proportion of time spent with small ruminants, the scores increased by 0.2%. Cognitive level (the revised Bloom’s level) also had an effect in that for each level increase in difficulty, the scores decreased by 5.24%. Congress attendance was not a significant variable in the model.

Wrenn and Wrenn (2009) discuss the importance of CPD in education. Veterinarians have to comply with CPD requirements to maintain registration with the South African Veterinary Council (SAVC) to be eligible to continue practising. This gathering of information and practice in the form of CPD may also have contributed to the higher scores obtained by the veterinarians, and therefore, it may not be practical experience alone that contributed to higher scores. It was not assessed whether the CPD points collected by the veterinarians in this study were focussed on production animals, although the participants were rural practitioners at the time of the study. This can be the focus of further studies. It can be noted that some of the participants had moved to companion animal practice from a previously mixed practice, and this could account for the discrepancy in time spent with ruminants, number of years’ experience and scores. Wrenn and Wrenn (2009) concluded that the balance of theory and practical is best achieved over the course of a curriculum, rather than within individual modules. What is interesting to note though is that congress attendance (considered a form of CPD) did not have an effect on the theoretical knowledge in the way that practical experience did and thus did not feature in the model in Table 3. This can be because congresses are presented as lectures. It could be investigated in future whether congresses with more practical components have a greater influence on cementing theoretical knowledge.

An additional finding of the study was that the use of acronyms can be confusing during questions, for example, PEM can be used as an acronym for both protein-energy malnutrition and Polioencephalomalacia. The confusion caused by acronyms was noted by a few of the practitioners who participated in the study. It is therefore recommended not to use acronyms in assessments.

Although the veterinarians received the questions in a set order, questions in a computer-based assessment are usually randomised per student to prevent students from comparing answers with their peers whilst in the assessment environment. Practitioners were less likely to refer to each other’s answers, as there are time constraints in practice, and this was not a high-stakes assessment for the practitioners. There was no reward or incentive for the veterinarians’ participation as the results remained anonymous, and there was no competition amongst them. It may be possible that the order in which questions are presented could influence the way in which students interpret and thus answer questions (McClendon & O’Brien 1988). In designing a written paper, lecturers will often begin the paper with a relatively easy question that most students will be able to answer. The next question may be a question that borderline students have some difficulty with, but that the majority of the class may still answer correctly, and then questions aimed at distinction candidates will be interspersed. This gives the candidates confidence (McClendon & O’Brien 1988). When faced with a question that is aimed at distinction candidates as a first question, some students may feel despondent or intimidated, and this can potentially affect the results of the test or exam. However, as the student group was much larger than the veterinarian group, the effect may be diluted. This is a topic for future studies, although it is recommended that randomisation of questions is achieved in blocks according to the level of the revised Bloom’s taxonomy to avoid this phenomenon.

Practitioners tended to rather leave questions blank than give an incorrect answer (dealt with as missing data according to Dohoo et al. 2003), whereas students seemed to be more comfortable with randomly allocating an answer as time allocated for the assessment ran out. Anecdotal evidence suggests that students will often do this hoping that they could guess a correct answer and thus improve their mark. Whether or not negative marking should be performed, to prevent guessing, is a topic of other research.

From this study, it can be seen that the greatest difference in scores was between the veterinarians and the students. Once qualified, the number of years of clinical practical experience had the greatest effect on the scores obtained for the veterinarians. Time spent with ruminants clearly also had a significant effect on their scores. These two factors determined which of the veterinarians performed the best in the assessment and that working for a number of years in a specific discipline will provide the best support for theoretical knowledge. Therefore, the recommendation is that clinical practical exposure should be encouraged from the first year of study in all possible clinical fields. It would have been interesting for this study to have the same students complete the assessment again having completed the clinical practical component of the degree. This should be included in further research.

## Conclusion

It is concluded that clinical practical experience has a positive effect on theoretical knowledge, particularly at a higher cognitive level. The type of clinical practical experience and where such experience is included in a curriculum need further research.
